# YIPF5 is an essential host factor for porcine epidemic diarrhea virus double-membrane vesicle formation

**DOI:** 10.1128/jvi.00320-25

**Published:** 2025-05-27

**Authors:** Liang Guo, Xiaoyue Duan, Jin Li, Zhuolin Hao, Yuhai Bi, Yuhai Chen, Xuguang Du, Sen Wu

**Affiliations:** 1 Sanya Institute of China Agricultural University, Sanya, China; 2 State Key Laboratory of Animal Biotech Breeding, College of Biological Sciences, China Agricultural University 98442, Beijing, China; 3 CAS Key Laboratory of Pathogen Microbiology and Immunology, Institute of Microbiology, Core-facility for Biosafety and Laboratory Animal, Center for Influenza Research and Early-warning (CASCIRE), CAS-TWAS Center of Excellence for Emerging Infectious Diseases (CEEID), Chinese Academy of Sciences 85387, Beijing, China; 4 University of the Chinese Academy of Sciences 74519, Beijing, China; 5 Beijing Research Center for Respiratory Infectious Diseases, Beijing Key Laboratory of Surveillance, Early Warning and Pathogen Research on Emerging Infectious Diseases, Beijing, China; Loyola University Chicago - Health Sciences Campus, Maywood, Illinois, USA

**Keywords:** porcine epidemic diarrhea virus, CRISPR/Cas9, YIPF5, DMVs, host factor

## Abstract

Porcine epidemic diarrhea virus (PEDV) is a major coronavirus in swine, causing substantial economic losses in the industry. To deepen our understanding of the PEDV–host cell interactions, we performed whole-genome CRISPR/Cas9 screens on porcine IPEC-J2 and IPI-2I cell lines to identify key host factors essential for PEDV infection. Our study identified the Yip family 5 (YIPF5) protein as a critical host factor, where its knockout suppressed PEDV infection by specifically affecting the virus replication stage. YIPF5 interacts with viral non-structural protein (nsp) 3, 4, and 6, facilitating the formation of double-membrane vesicles (DMVs), essential for replication organelle biogenesis. The knockout of YIPF5 interferes with the interaction between nsp3 and nsp4, consequently impacting the formation of DMVs mediated by these proteins. These findings establish YIPF5 as a key host factor involved in DMV formation during PEDV infection, highlighting its potential as a therapeutic target.

**IMPORTANCE:**

Coronaviruses pose serious health threats to both humans and animals. Identifying host genes critical for porcine epidemic diarrhea virus (PEDV) infection can uncover new therapeutic targets and enhance our understanding of coronavirus pathogenesis. In this study, we conducted genome-scale CRISPR/Cas9 screens in two porcine cell lines (IPEC-J2 and IPI-2I) and identified YIPF5 as an essential host factor for PEDV replication. Our results demonstrate that YIPF5 plays a pivotal role in the formation of PEDV-induced double-membrane vesicles (DMVs), which are crucial for viral replication. These findings shed new light on the molecular mechanisms of PEDV and suggest YIPF5 as a therapeutic target.

## INTRODUCTION

Coronaviruses (CoVs) are enveloped, single-stranded, positive-sense RNA viruses that infect both humans and various animals. The four principal genera of CoVs encompass α, β, γ, and δ. Severe acute respiratory syndrome coronavirus 2 (SARS-CoV-2), a β-CoV, causes upper respiratory diseases, fever, and severe pneumonia in humans, leading to a global pandemic in recent years (1–3). Porcine epidemic diarrhea virus (PEDV), an α-CoV, induces diarrhea, vomiting, and dehydration in piglets, with a mortality rate of up to 100% in young piglets, causing significant economic losses in the swine industry (4, 5). Currently, virus prevention and treatment strategies primarily rely on vaccines. However, the virus’s persistent mutability poses a formidable challenge to these efforts. The advent of CRISPR/Cas9 gene-editing technology has introduced new approaches for virus prevention and control. This technology has been widely applied in various research fields, including the screening of viral host factors (6, 7), identification of drug targets (8, 9), and development of disease-resistant breeding programs (10, 11). Previous CRISPR/Cas9 screens for PEDV host factors have utilized non-porcine cell lines, such as Vero E6 and HEK293T (12, 13). Our study innovates by employing pig-derived cell lines (IPEC-J2 and IPI-2I) for PEDV screens.

CoVs initiate infection by binding to host cell receptors via their spike (S) protein, which is subsequently cleaved by host transmembrane proteases to facilitate membrane fusion (14–16). Following this, the viral RNA is released into the cytoplasm, where the first two open-reading frames (ORFs), 1a and 1b, are translated into polyproteins and subsequently cleaved by viral proteases into 16 non-structural proteins (nsps) (17, 18). These 16 nsps include 13 cytosolic proteins and three transmembrane proteins, namely, nsp3, nsp4, and nsp6. Nsp3, nsp4, and nsp6 are crucial for the formation of the CoVs replication organelle (RO), double-membrane vesicles (DMVs) (19–21). Specifically, the co-expression of nsp3 and nsp4 is both necessary and sufficient to induce DMV formation, while nsp6 plays a role in stabilizing the connection between the endoplasmic reticulum (ER) membranes and DMVs (19, 20). DMVs provide a safe environment for viral replication, allowing the virus to evade the host’s innate immune response.

The formation of SARS-CoV-2 DMVs is orchestrated by various essential host factors, such as TMEM41B, VMP1, REEP5, TRAM1, RTN3, and RTN4 (22–24). VMP1 is recruited to nsp3/4-positive structures via its luminal loop. In VMP1 knockout (KO) cells, DMV closure is impaired, although the nsp3–nsp4 binding interaction remains unaffected. Conversely, TMEM41B deficiency significantly hampers the formation of nsp3/4 complexes. Both VMP1 and TMEM41B regulate phosphatidylserine distribution, functioning as scramblases (22). REEP5 and TRAM1 interact with nsp3 at the membrane of the RO, facilitating RO biogenesis and enhancing viral replication (23). Additionally, ER morphogenic proteins, specifically reticulon-3 (RTN3) and RTN4, play a pivotal role in promoting DMV formation. Mechanistically, the membrane-embedded reticulon homology domain (RHD) within RTNs supports viral replication and physically interacts with nsp3 and nsp4 (24). However, the specific roles of host factors in PEDV DMV formation remain largely unexplored and warrant further investigation.

In this study, we identified key host factors essential for PEDV infection through whole-genome KO screens conducted in IPEC-J2 and IPI-2I cell lines. Notably, we discovered that YIPF5, a member of the Yip family, interacts with PEDV nsp3, nsp4, and nsp6, actively participating in the formation of PEDV DMVs. The knockout of YIPF5 significantly reduced DMV formation, thereby diminishing PEDV replication. These findings underscore the potential importance of YIPF5 as a promising therapeutic target for the effective prevention and treatment of PEDV infections.

## RESULTS

### Genome-scale CRISPR/Cas9 knockout screens identify host factors critical for PEDV infection

To identify key host factors involved in PEDV infection, we conducted two genome-scale CRISPR/Cas9 KO library screens in porcine intestinal epithelial cell lines, IPEC-J2 and IPI-2I, using the PEDV CV777 strain. We infected IPEC-J2 and IPI-2I cell lines with lentiviruses carrying Cas9 and the pig KO library, which includes 136,106 sgRNAs targeting specific genes and 2,000 non-targeting sgRNAs, at a multiplicity of infection (MOI) of 0.3. After 3 d of puromycin selection, the cells were allowed to recover for an additional 7 d.

**Fig 1:**
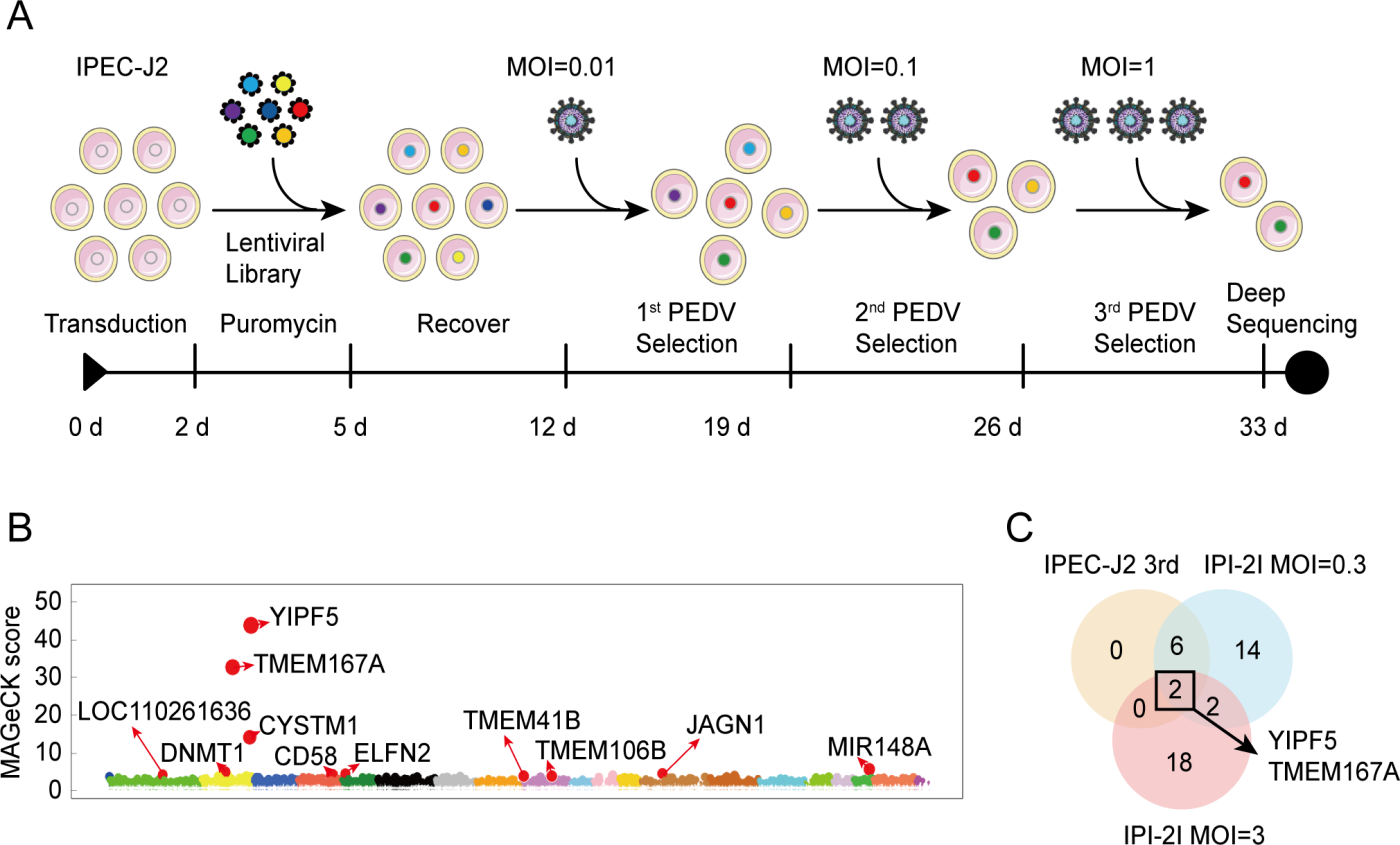
Genome-scale CRISPR/Cas9 knockout screens identify host factors required for PEDV infection. (**A**) Flowchart for identification of PEDV essential host factors using genome-scale CRISPR/Cas9 KO library screens in IPEC-J2 cell lines. The IPEC-J2 KO library was subjected to three rounds of PEDV infection with escalating MOIs (0.01, 0.1, and 1). Surviving cells from the third round of virus challenge were selected, PCR amplified, and subjected to deep sequencing of sgRNAs. (**B**) MAGeCK analyses of the third round of PEDV screens in the IPEC-J2 KO library. (**C**) Venn diagram illustrating the overlap of differential genes identified in three screens. MOI, multiplicity of infection.

In the IPEC-J2 KO library, we conducted three sequential rounds of PEDV CV777 infection with escalating MOIs (0.01, 0.1, and 1). After the final round, the surviving cells were collected, amplified, and subjected to sgRNA sequencing (Fig. 1A). The IPI-2I KO library was infected with PEDV CV777 at two distinct MOIs (0.3 and 3), and the surviving cells were also sequenced for sgRNAs.

We employed MAGeCK and *Z*-score analyses to compare sgRNAs from surviving cells to those from an uninfected cell library, aiming to detect significant differences. In the IPEC-J2 KO library screen, MAGeCK analyses revealed significant enrichment of 622 genes (*P* < 0.05) (Table S1). Notably, TMEM41B and TMEM106B were identified, which have been implicated in previous coronavirus studies (25–27) (Fig. 1B; Table S1). *Z*-score analyses (*Z*-score > 2) identified eight candidate factors from the IPEC-J2 KO library screen, including YIPF5, TMEM167A, CYSTM1, IER3IP1, PHYHD1, ACBD6, and two unknown factors (LOC106510126 and LOC110260313) (Fig. S1; Table S1). The IPI-2I KO library screens also identified YIPF5 and TMEM167A as significant factors (Fig. 1C; Table S2). These findings confirm the effectiveness and reliability of our genome-scale CRISPR/Cas9 KO library screens in both IPEC-J2 and IPI-2I cell lines, highlighting the pivotal role of these host factors in PEDV infection.

### YIPF5 is an essential host factor required for PEDV infection

Following our promising screen results, we established monoclonal YIPF5 KO IPEC-J2 cell lines using CRISPR/Cas9 technology. To ensure a comprehensive functional gene knockout, we utilized two sgRNAs to target and eliminate substantial genomic regions within YIPF5 (Fig. S2A). Comparative cell proliferation assays using CCK8 indicated no significant differences between wild-type (WT) and YIPF5 KO IPEC-J2 cell lines (Fig. S2B).

**Fig 2:**
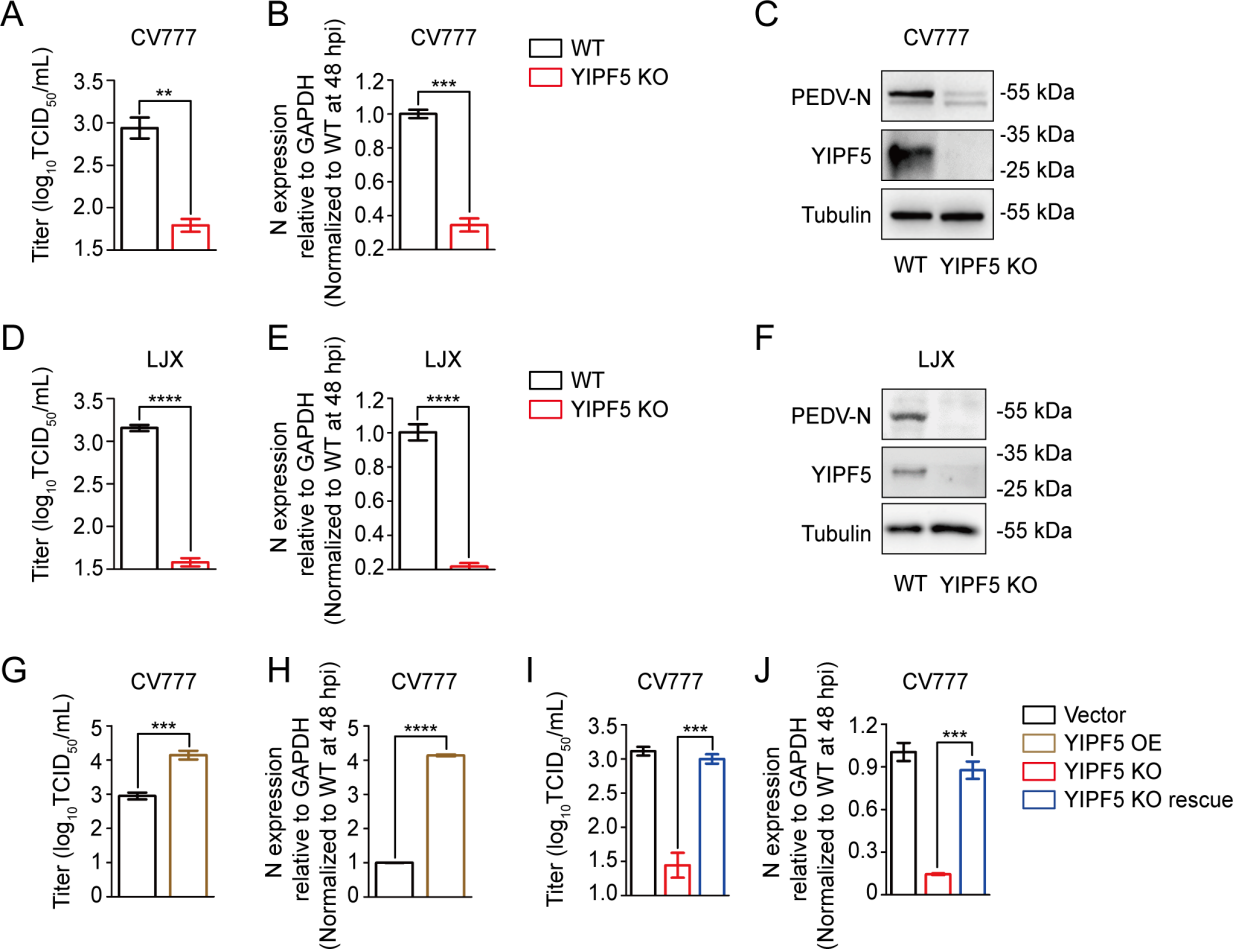
YIPF5 is an essential host factor required for PEDV infection. (**A, D**) Quantification of virus infectivity (TCID_50_) in cultural supernatant of WT and YIPF5 KO IPEC-J2 cell lines infected with PEDV CV777 (**A**) or LJX (**D**) at 48 h (MOI = 1). (**B, E**) RT-qPCR assay for determining the relative mRNA expression of N protein in WT and YIPF5 KO IPEC-J2 cell lines infected with PEDV CV777 (**B**) or LJX (**E**) at 48 h (MOI = 1). (**C, F**) Western blot assays for detecting the PEDV N protein in WT and YIPF5 KO IPEC-J2 cell lines infected with PEDV CV777 (**C**) or LJX (**F**) at 48 h (MOI = 1). Tubulin served as the internal control protein. (**G**) TCID_50_ in cultural supernatant of vector and overexpression of YIPF5-Flag IPEC-J2 cell lines infected with PEDV CV777 at 48 h (MOI = 1). (**H**) RT-qPCR assay for determining the relative mRNA expression of N protein in vector and overexpression of YIPF5-Flag IPEC-J2 cell lines infected with PEDV CV777 at 48 h (MOI = 1). (**I**) TCID_50_ in cultural supernatant of vector, YIPF5 KO, and YIPF5 knockout rescued (KO rescue) IPEC-J2 cell lines infected with PEDV CV777 at 48 h (MOI = 1). (**J**) RT-qPCR assay for determining the relative mRNA expression of PEDV CV777 N protein in vector, YIPF5 KO, and YIPF5 KO rescue IPEC-J2 cell lines infected with PEDV CV777 at 48 h (MOI = 1). WT, wild type; KO, knockout; MOI, multiplicity of infection; N, nucleocapsid. **P < 0.01, ***P < 0.001, ****P < 0.0001. *P* values were determined by two-sided Student’s *t*-test. Data are representative of at least three independent experiments.

We then challenged WT and YIPF5 KO IPEC-J2 cell lines with PEDV CV777 (subtype G1) and LJX (subtype G2), respectively. The absence of YIPF5 resulted in a significant decrease in viral titers for both strains (Fig. 2A and D). RT-qPCR analyses further revealed a reduced relative mRNA expression of PEDV nucleocapsid (N) protein in YIPF5 KO compared to WT IPEC-J2 cell lines (Fig. 2B and E). Western blot analyses confirmed these findings, demonstrating lower levels of PEDV-encoded N protein in YIPF5 KO IPEC-J2 cell lines after infection with PEDV CV777 and LJX (Fig. 2C and F).

Additionally, the overexpression of YIPF5-Flag in IPEC-J2 cell lines followed by infection with PEDV CV777 resulted in increased viral titers and relative mRNA expression of N protein compared to control (Fig. 2G and H; Fig. S3). To further validate the indispensable role of YIPF5 in PEDV infection, we rescued YIPF5-Flag expression in YIPF5 KO IPEC-J2 cell lines, ensuring a stable expression (Fig. S3). As anticipated, the infectivity of PEDV was restored in the rescued YIPF5 KO IPEC-J2 cell lines compared to control (Fig. 2I and J). Collectively, these results underscore YIPF5 as an essential host factor for PEDV infection.

To further investigate whether YIPF5 plays a role in the infection process of other CoVs, we examined the replication of human coronavirus OC43 (HCoV-OC43) and porcine deltacoronavirus (PDCoV) in WT and YIPF5 KO ACE2-Vero E6 cell lines (Fig. S2). RT-qPCR analyses revealed that the replication of both HCoV-OC43 and PDCoV was reduced following the knockout of YIPF5 (Fig. S4A and B). These findings suggest that YIPF5 may play a significant role in the infection process of CoVs.

### Knockout of YIPF5 does not affect PEDV adsorption and internalization

**Fig 3:**
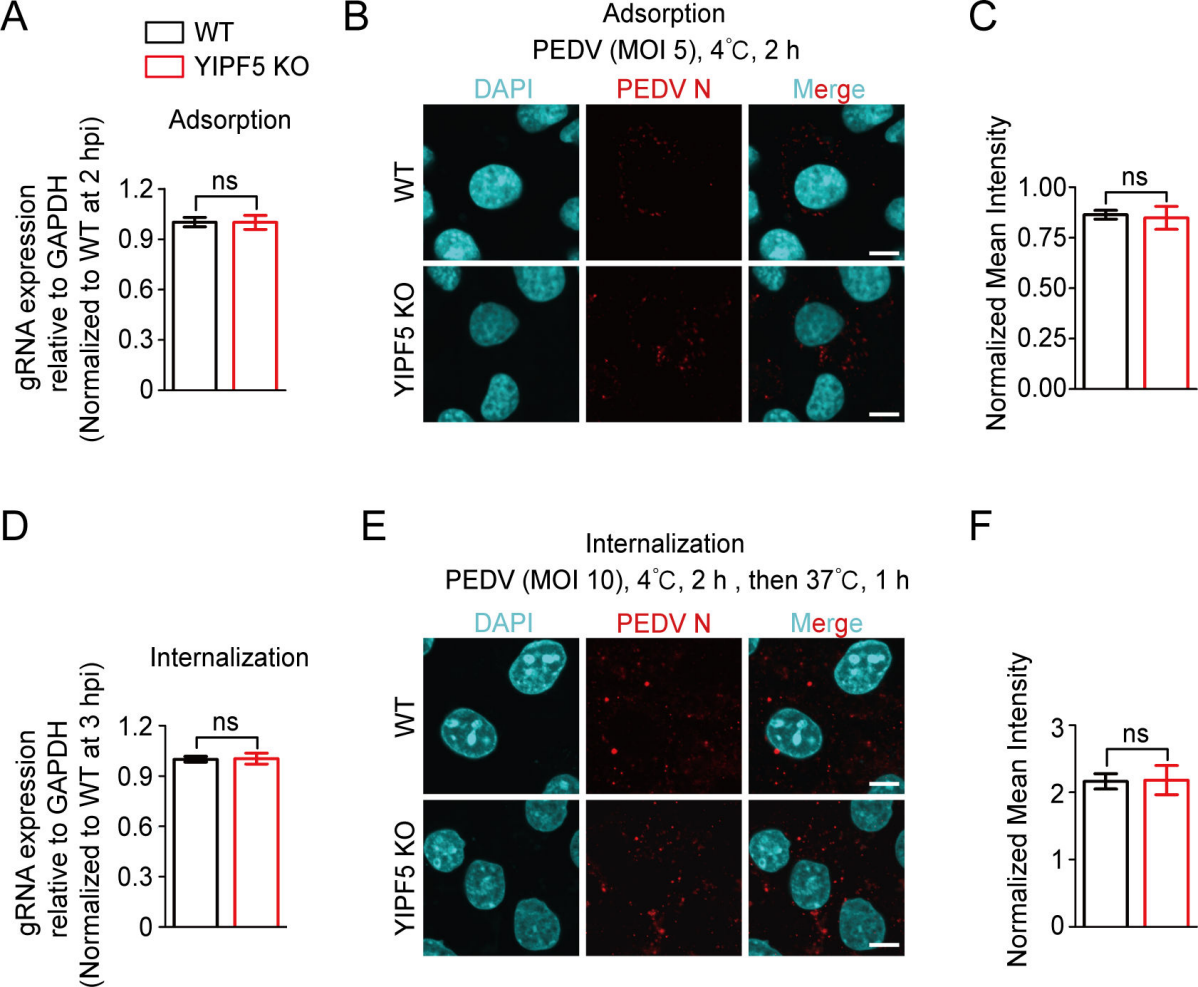
Knocking out YIPF5 does not affect PEDV adsorption and internalization. (**A**) Assessment of PEDV adsorption activity in WT and YIPF5 KO IPEC-J2 cell lines by RT-qPCR assay. WT and YIPF5 KO IPEC-J2 cell lines were incubated with PEDV CV777 at 4°C for 2 h (MOI = 5). (**B**) Immunofluorescence assays for detection of PEDV N protein in WT and YIPF5 KO IPEC-J2 cell lines incubated with PEDV CV777 at 4°C for 2 h (MOI = 5). Scale bars, 10 µm. (**C**) Normalized mean fluorescence intensity was measured using ImageJ. (**D**) Evaluation of the PEDV internalization stage in WT and YIPF5 KO IPEC-J2 cell lines by RT-qPCR assay. WT and YIPF5 KO IPEC-J2 cell lines were incubated with PEDV CV777 at 4°C for 2 h (MOI = 10) and transferred to 37°C for 1 h. (**E**) Immunofluorescence assays for detection of PEDV N protein in WT and YIPF5 KO IPEC-J2 cell lines incubated with PEDV CV777 at 4°C for 2 h (MOI = 10) and transferred to 37°C for 1 h. Scale bars, 10 µm. (**F**) Normalized mean fluorescence intensity was measured using ImageJ. WT, wild type; KO, knockout; MOI, multiplicity of infection; DAPI, 4’,6-diamidino-2-phenylindole; N, nucleocapsid; ns, not significant. *P* values were determined by two-sided Student’s *t*-test. Data are representative of at least three independent experiments.

To determine at which stage of PEDV infection YIPF5 functions, we investigated whether there were differences in the adsorption and internalization of PEDV in WT and YIPF5 KO IPEC-J2 cell lines. Initially, we assessed the adsorption rate of viral particles in WT and YIPF5 KO IPEC-J2 cell lines at 4°C for 2 h (MOI = 5). By monitoring the expression levels of PEDV genomic RNA (gRNA) as an indicator of viral adsorption, we found that both cell lines exhibited similar capabilities for virus particle adsorption (Fig. 3A). Additionally, the fluorescence intensity of PEDV N protein showed no significant differences between WT and YIPF5 KO IPEC-J2 cell lines (Fig. 3B and C). These results suggest that the knockout of YIPF5 does not affect the adsorption of viral particles.

Subsequently, we examined the viral internalization stage. WT and YIPF5 KO IPEC-J2 cell lines were challenged with PEDV at 4°C for 2 h and then transferred to 37°C for 1 h to facilitate viral internalization. RT-qPCR results showed that both cell lines exhibited similar internalization capabilities for viral particles (Fig. 3D). Immunofluorescence assays indicated that the fluorescence intensity of PEDV N protein in the cytoplasm of YIPF5 KO cells was comparable to those in WT cells (Fig. 3E and F). These results suggest that the knockout of YIPF5 does not affect the internalization of viral particles.

### The replication stage of PEDV was impaired in YIPF5 KO cells

**Fig 4:**
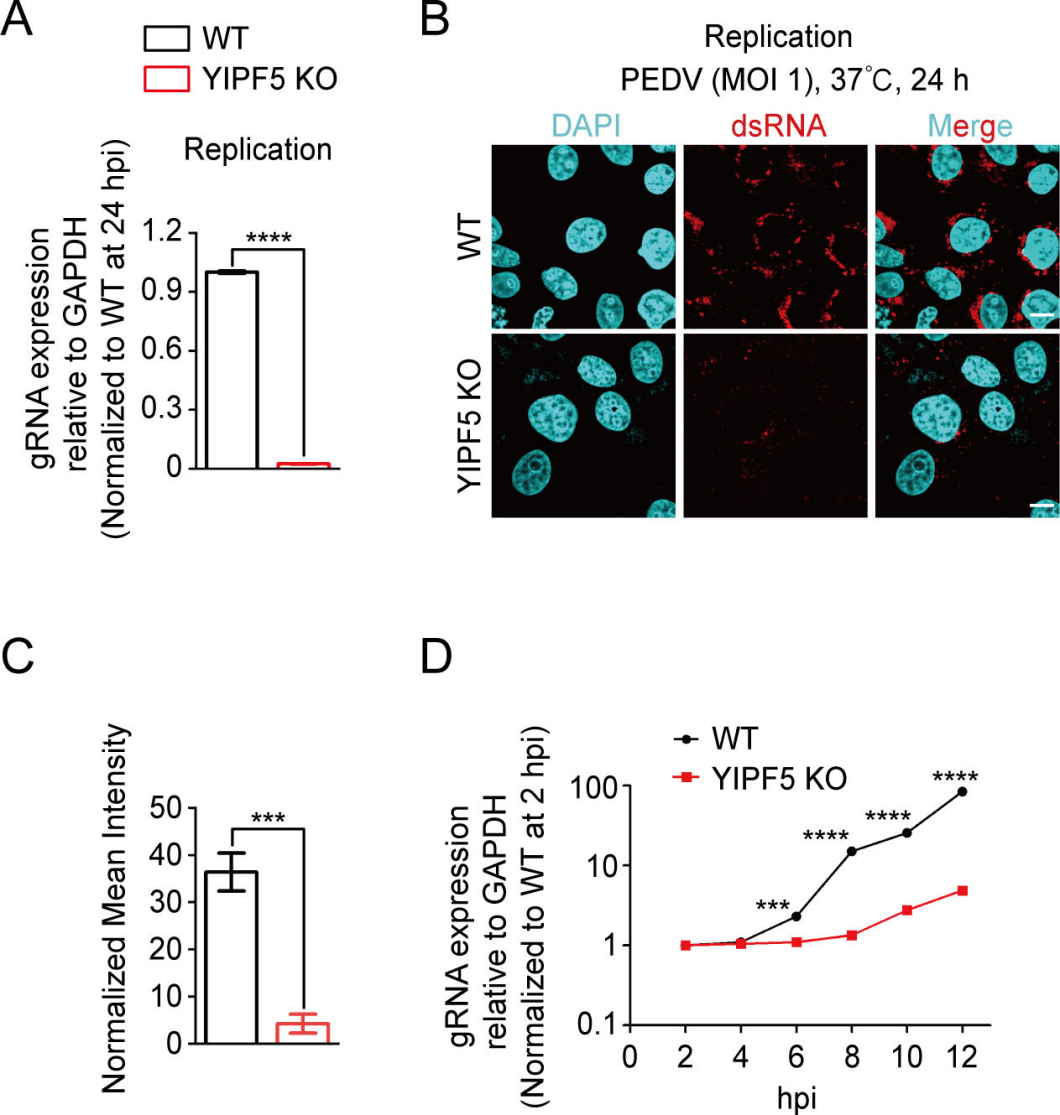
The replication stage of PEDV was impaired in YIPF5 KO IPEC-J2 cell lines. (**A**) Assessment of PEDV replication stage in WT and YIPF5 KO IPEC-J2 cell lines by RT-qPCR assay. WT and YIPF5 KO IPEC-J2 cell lines were incubated with PEDV CV777 at 37°C for 24 h (MOI = 1). (**B**) Immunofluorescence assays for detection of PEDV dsRNA in WT and YIPF5 KO IPEC-J2 cell lines infected with PEDV CV777 at 37°C for 24 h (MOI = 1). Scale bars, 10 µm. (**C**) Normalized mean fluorescence intensity was measured using ImageJ. (**D**) Viral growth curves within 12 h in WT and YIPF5 KO IPEC-J2 cell lines. WT, wild type; KO, knockout; MOI, multiplicity of infection; DAPI, 4’,6-diamidino-2-phenylindole; dsRNA, double-stranded RNA. ***P < 0.001, ****P < 0.0001. *P* values were determined by two-sided Student’s *t*-test. Data are representative of at least three independent experiments.

Because the knockout of YIPF5 does not affect the adsorption and internalization of PEDV, we next investigated whether the replication stage of PEDV was impacted. WT and YIPF5 KO IPEC-J2 cell lines were challenged with PEDV at 37°C for 24 h. RT-qPCR results showed that the relative mRNA expression level of PEDV N protein in YIPF5 KO IPEC-J2 cell lines was significantly lower compared to WT IPEC-J2 cell lines (Fig. 4A). Immunofluorescence assays also demonstrated that the fluorescence intensity of dsRNA in YIPF5 KO IPEC-J2 cell lines was reduced by approximately 85% compared to WT IPEC-J2 cell lines (Fig. 4B and C). To further determine when the replication of PEDV begins to be inhibited after the knockout of YIPF5, we monitored PEDV replication every 2 h within 12 h post-infection. The results showed that in the YIPF5 KO IPEC-J2 cell lines, compared to the WT cell lines, the replication of the virus was inhibited starting from 6 h post-PEDV infection (Fig. 4D). These findings suggest that YIPF5 is essential for the replication stage of PEDV.

### YIPF5 plays a pivotal role in the generation of PEDV DMVs

Previous studies have elucidated that upon infection of host cells by CoVs, the viruses hijack the host cell’s endomembrane system to form DMVs, which serve as sites for viral RNA replication (28, 29). YIPF5 (also known as YIP1A), a five-transmembrane protein, shuttles between the ER and the Golgi apparatus, playing a crucial role in maintaining the normal structure of the ER (30, 31). Given the endomembrane localization of YIPF5, it is plausible that it contributes to PEDV DMV formation. Previous studies have demonstrated that the formation of DMVs can be observed as early as 6 h post-infection with CoVs (32, 33). Our experimental results also revealed a significant reduction in viral replication in YIPF5 KO IPEC-J2 cell lines compared to WT IPEC-J2 cell lines within the first 6 h post-PEDV infection (Fig. 4D). This finding leads us to further suspect that YIPF5 is involved in the formation of DMVs.

**Fig 5:**
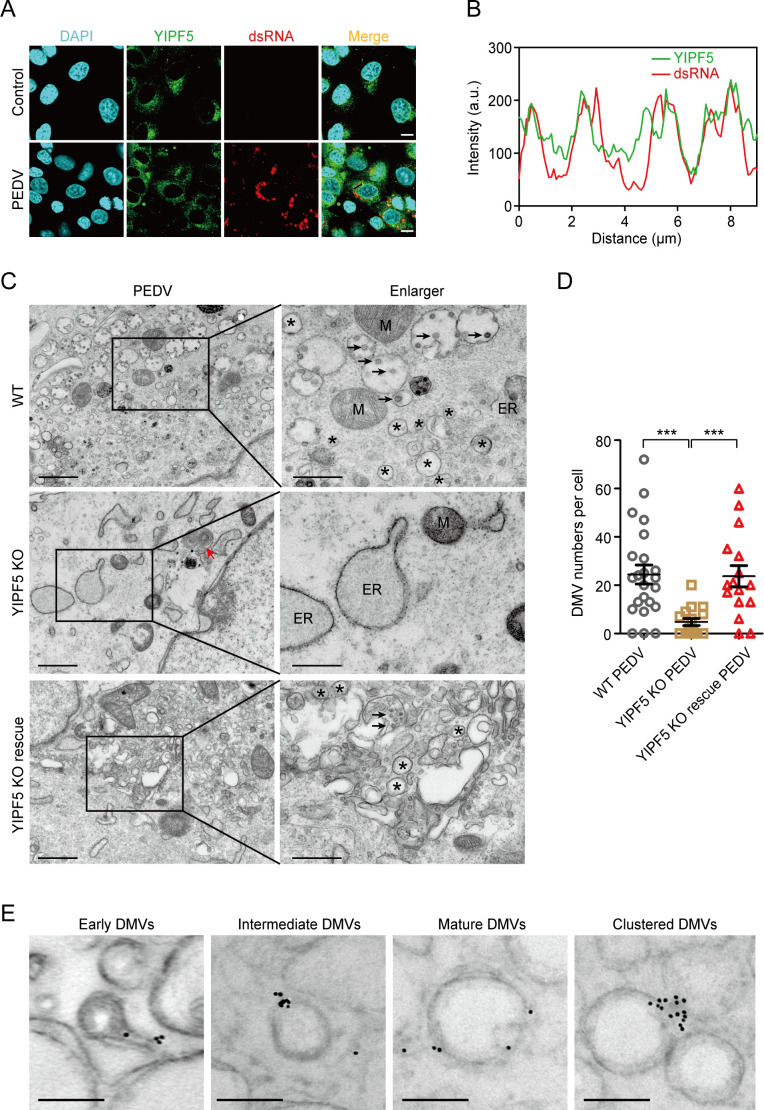
YIPF5 contributes to the formation of PEDV DMVs. (**A**) Analyses of the subcellular location of YIPF5 and dsRNA in IPEC-J2 cell lines uninfected (above) or infected with PEDV after 48 h (MOI = 1) (bottom). The YIPF5 (indicated in green) was co-localized with dsRNA (indicated in red). Scale bar, 10 µm. (**B**) Measure of the fluorescence intensity of YIPF5 and dsRNA at the same location in panel A (PEDV-Merge, indicated by the black line). (**C**) Representative transmission electron microscope images depicting PEDV-induced DMVs (indicated by asterisks) in WT, YIPF5 KO, and YIPF5 KO rescue IPEC-J2 cell lines infected with PEDV after 48 h (MOI = 1). The black arrows indicate viral particles. The red arrow indicates stacked whorl. Scale bar, 1 µm (left) or 500 nm (right). (**D**) Quantification of the number of DMVs in WT, YIPF5 KO, and YIPF5 KO rescue IPEC-J2 cell lines infected with PEDV after 48 h (MOI = 1). *N* ≥ 16 cells for each cell line were calculated. (**E**) Representative immunoelectron microscope images depicting DMVs in IPEC-J2 cell lines infected with PEDV after 48 h (MOI = 1) and location of YIPF5. Scale bar, 200 nm. WT, wild type; KO, knockout; MOI, multiplicity of infection; DAPI, 4’,6-diamidino-2-phenylindole; dsRNA, double-stranded RNA; M, mitochondria; ER, endoplasmic reticulum. ***P < 0.001. *P* values were determined by two-sided Student’s *t*-test. Data are representative of at least three independent experiments.

Confocal assay results revealed that YIPF5 exhibits a diffuse ER pattern and co-localizes with dsRNA upon PEDV infection (Fig. 5A and B). We conducted transmission electron microscopy (TEM) assays to assess the capability of PEDV DMV formation in WT, YIPF5 KO, and YIPF5 KO rescue IPEC-J2 cell lines. Notably, we observed typical DMVs (with a diameter of approximately 100–400 nm) in WT IPEC-J2 cell lines post-PEDV infection. However, in YIPF5 KO IPEC-J2 cell lines, the number of DMVs observed was extremely low, and they were almost undetectable. After complementation of YIPF5, the formation of DMVs was restored (Fig. 5C). We randomly selected 16–23 cells from PEDV-infected WT, YIPF5 KO, and YIPF5 KO rescue IPEC-J2 cell lines to count the number of DMVs. In the YIPF5 KO IPEC-J2 cell lines, the number of DMVs was significantly lower compared to the WT IPEC-J2 cell lines. However, upon restoration of YIPF5, the quantity of DMVs returned to levels comparable to those observed in the WT cells (Fig. 5D). To further explore the relationship between YIPF5 and DMVs, we conducted immunoelectron microscopy assays. The results showed that YIPF5 positive signals were observed in the early stage (diameter approximately 100 nm), intermediate stage (diameter approximately 150 nm), and mature stage (diameter approximately 200 nm) of DMV formation. Interestingly, YIPF5 positive signals were also detected between clustered DMVs (Fig. 5E). Hence, YIPF5 plays a pivotal role in the generation of PEDV DMVs.

### YIPF5 interacts with PEDV nsp3, nsp4, and nsp6

**Fig 6:**
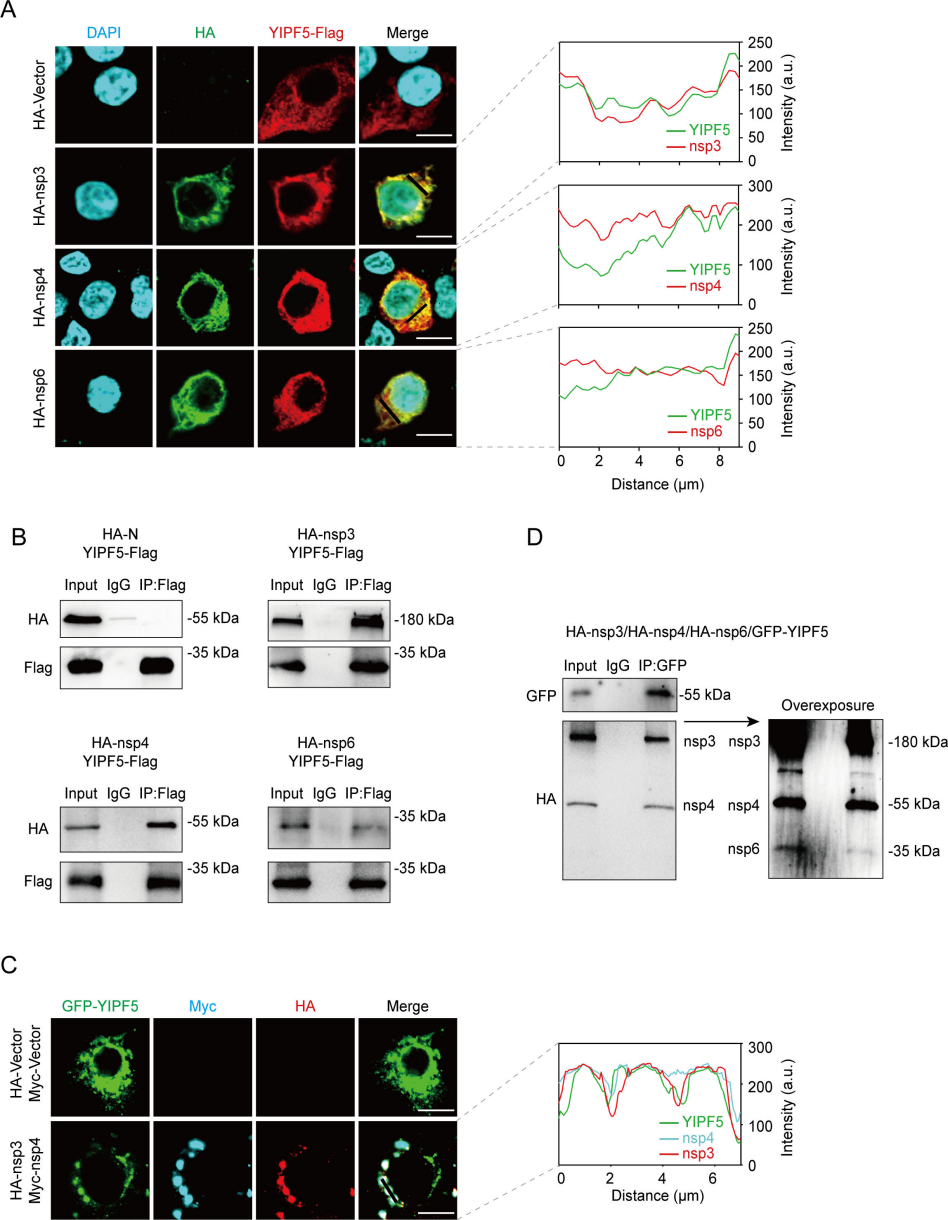
YIPF5 interacts with PEDV non-structural proteins 3, 4, and 6. HEK293T cells were co-transfected with YIPF5-Flag and PEDV HA-nsp3, HA-nsp4, or HA-nsp6 after 24 h for confocal assays (**A**) and co-IP assays (**B**). (**A**) Confocal fluorescence microscopy analyses of the co-localization of YIPF5-Flag (indicated in red) and HA-nsp3, HA-nsp4, or HA-nsp6 (indicated in green) in HEK293T cells. Measure of the fluorescence intensity of YIPF5 and nsp3, nsp4, or nsp6 at the same location (indicated by the black lines). Scale bar, 10 µm. (**B**) Protein interactions were analyzed by immunoprecipitation with anti-Flag beads and immunoblotting with anti-Flag and anti-HA. The co-transfection of YIPF5-Flag and PEDV HA-N served as the control group. (**C**) HEK293T cells were co-transfected with GFP-YIPF5, HA-nsp3, and Myc-nsp4 after 24 h for confocal assays. Confocal fluorescence microscopy analyses of the co-localization of GFP-YIPF5 (indicated in green), HA-nsp3 (indicated in red), and Myc-nsp4 (indicated in cyan) in HEK293T cells. Measure of the fluorescence intensity of YIPF5, nsp3, and nsp4 at the same location (indicated by the black lines). Scale bar, 10 µm. (**D**) HEK293T cells were co-transfected with GFP-YIPF5, HA-nsp3, HA-nsp4, and HA-nsp6 after 24 h for co-IP assays. Protein interactions were analyzed by immunoprecipitation with anti-GFP beads and immunoblotting with anti-GFP and anti-HA. DAPI, 4’,6-diamidino-2-phenylindole.

Previous research has established that the nsp3 and nsp4 are necessary and sufficient to induce the formation of DMVs (19, 22). Nsp6 facilitates the zipping of ER membranes and the organization of DMV clusters via its oligomerization and an amphipathic helix (20). To verify whether YIPF5 interacts with nsp3, nsp4, or nsp6, we co-transfected HEK293T cells with YIPF5-Flag plasmids and HA-nsp3, HA-nsp4, or HA-nsp6 plasmids individually. We then performed confocal assays and co-immunoprecipitation (co-IP) assays to assess potential protein–protein interactions. Confocal assays revealed robust co-localization of YIPF5 with nsp3, nsp4, and nsp6, providing initial evidence for their interactions (Fig. 6A). Co-IP assays further confirmed that YIPF5 could precipitate nsp3, nsp4, and nsp6, reinforcing the notion that YIPF5 physically interacts with these proteins (Fig. 6B).

Considering that nsp3 or nsp4 expressed alone cannot form DMVs, we co-transfected GFP-YIPF5, HA-nsp3, and Myc-nsp4 into HEK293T cells, and performed co-localization analyses. When GFP-YIPF5 was expressed alone, it exhibited a dispersed distribution. However, upon co-expression with HA-nsp3 and Myc-nsp4, GFP-YIPF5 formed puncta together with nsp3 and nsp4 (Fig. 6C). We also performed co-IP assays in HEK293T cells co-transfected with GFP-YIPF5, HA-nsp3, HA-nsp4, and HA-nsp6. Nsp3, nsp4, and nsp6 were all pulled down by YIPF5 (Fig. 6D). These results suggest that YIPF5 may be involved in the formation of DMVs through its interaction with nsp3, nsp4, and nsp6.

### Knockout of YIPF5 disrupts the interaction between nsp3 and nsp4, thereby affecting the formation of DMVs driven by nsp3 and nsp4

**Fig 7:**
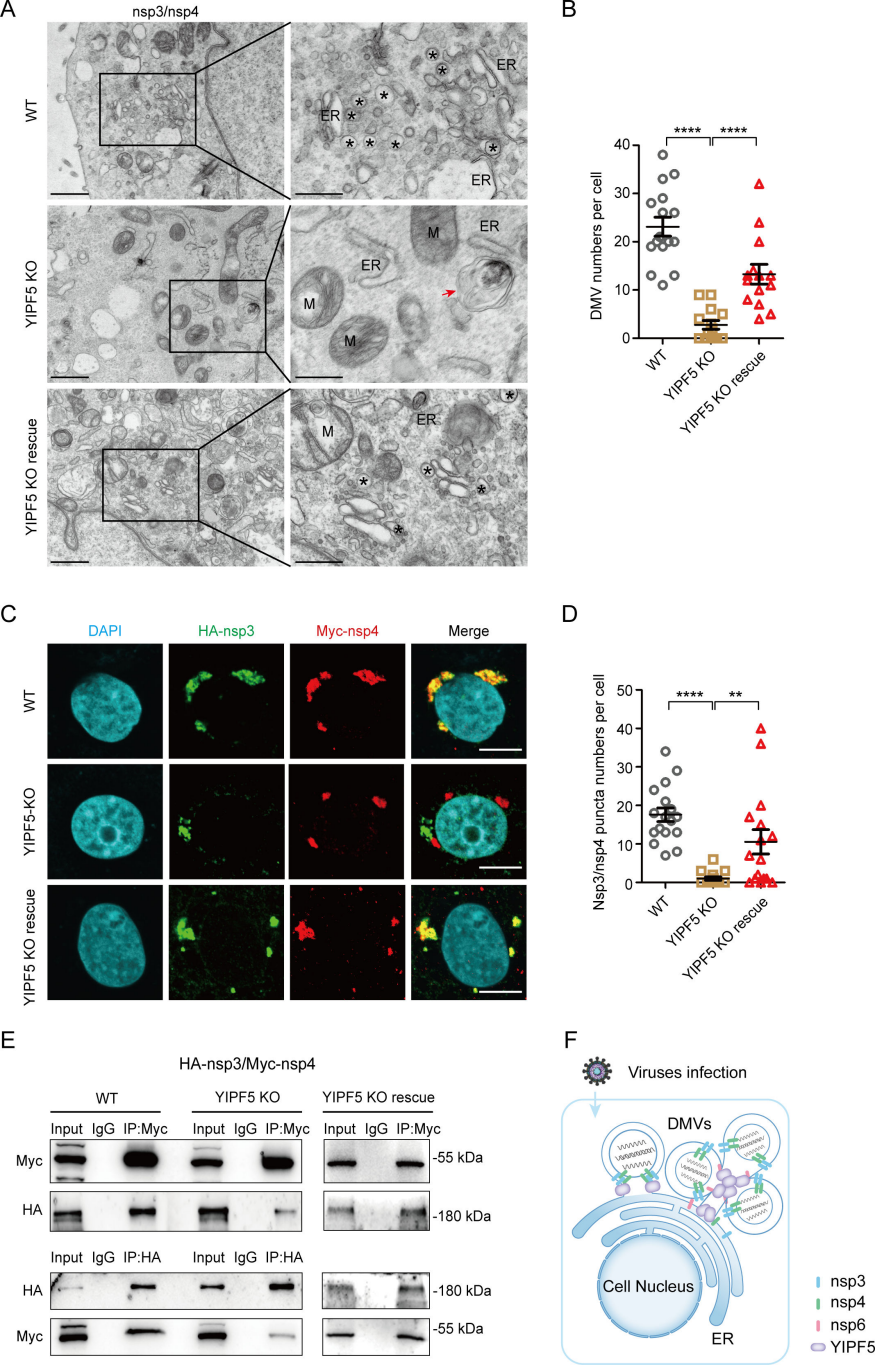
Knocking out YIPF5 affects the interaction between nsp3 and nsp4, thereby impacting the formation of DMVs driven by nsp3 and nsp4. (**A**) Representative transmission electron microscope images depicting nsp3/nsp4-induced DMVs (indicated by asterisks) in WT, YIPF5 KO, and YIPF5 KO rescue IPEC-J2 cell lines. The red arrow indicates the zippered structure. Scale bar, 1 µm (left) or 500 nm (right). (**B**) Quantification of the number of DMVs driven by nsp3 and nsp4 in WT, YIPF5 KO, and YIPF5 KO rescue IPEC-J2 cell lines. *N* ≥ 14 cells for each cell line were calculated. (**C**) WT, YIPF5 KO, and YIPF5 KO rescue IPEC-J2 cell lines were co-transfected with HA-nsp3 and Myc-nsp4 after 24 h for confocal assays. Confocal fluorescence microscopy analyses of the co-localization of HA-nsp3 (indicated in green) and Myc-nsp4 (indicated in red) in WT, YIPF5 KO, and YIPF5 KO rescue IPEC-J2 cell lines. Scale bar, 10 µm. (**D**) The number of nsp3/nsp4 puncta was quantified. *N* ≥ 16 cells for each cell line were calculated. (**E**) WT, YIPF5 KO, and YIPF5 KO rescue IPEC-J2 cell lines were co-transfected with HA-nsp3 and Myc-nsp4 after 24 h for co-IP assays. Protein interactions were analyzed by immunoprecipitation with anti-Myc beads or anti-HA beads and immunoblotting with anti-Myc and anti-HA. (**F**) Schematic diagram of YIPF5 involvement in PEDV DMV formation. WT, wild type; KO, knockout; DAPI, 4’,6-diamidino-2-phenylindole; M, mitochondria; ER, endoplasmic reticulum. **P < 0.01, ****P < 0.0001. *P* values were determined by two-sided Student’s *t*-test. Data are representative of at least three independent experiments.

Previous studies have identified SARS-CoV-2 nsp3 and nsp4 as the minimal components required for the formation of DMVs (34). We co-transfected PEDV nsp3 and nsp4 into WT, YIPF5 KO, and YIPF5 KO rescue IPEC-J2 cell lines. The TEM assays demonstrated that nsp3 and nsp4 can induce DMVs (with a diameter of approximately 100–200 nm) in WT IPEC-J2 cell lines (Fig. 7A), suggesting that the co-expression of nsp3 and nsp4 is sufficient to induce DMV formation. In contrast, the formation of DMVs was significantly suppressed in YIPF5 KO IPEC-J2 cell lines. Nevertheless, upon complementation of YIPF5, both the formation and quantity of DMVs mediated by nsp3 and nsp4 were successfully restored (Fig. 7A and B). Interestingly, we observed a zippered structure in proximity to ER in YIPF5 KO cells (Fig. 7A). HA-nsp3 and Myc-nsp4 were transfected into WT, YIPF5 KO, and YIPF5 KO rescue IPEC-J2 cell lines. Confocal assays showed that nsp3 and nsp4 formed puncta in WT cells, while the number of puncta formed by nsp3 and nsp4 was significantly reduced in YIPF5 KO cells. Upon supplementation of YIPF5, the ability of nsp3 and nsp4 to form puncta was reinstated (Fig. 7C and D). To further investigate the effect of YIPF5 knockout on the interaction between nsp3 and nsp4, we co-transfected HA-nsp3 and Myc-nsp4 into WT, YIPF5 KO, and YIPF5 KO rescue IPEC-J2 cell lines. Co-IP assays revealed that the interaction between nsp3 and nsp4 was reduced in YIPF5 KO cells compared to WT cells. After complementation of YIPF5, the interaction between nsp3 and nsp4 was reestablished (Fig. 7E). These results collectively indicate that YIPF5 plays a crucial role in the formation of DMVs mediated by nsp3 and nsp4, likely functioning as an organizer.

## DISCUSSION

CoVs pose a significant threat to global health, necessitating comprehensive studies to elucidate their replication mechanisms. These insights are crucial for identifying new therapeutic targets to effectively treat CoV infection. Recent research used a whole-genome KO library in Vero E6 cell lines to identify host factors crucial for PEDV infection. TRIM2 and SLC35A1 were identified as significant host factors in PEDV infection. Knocking out SLC35A1 increased ADAM17 expression, which enhanced the removal of pAPN and ACE2 extracellular domains, reducing PEDV adhesion (12). In another study, human HEK293T cells were employed as the screen cell lines for PEDV, revealing that PEDV enhances viral endocytosis and replication through mitochondrial apoptosis by activating the PKCθ-BCL-2 ovarian killer (BOK) axis (13). Given that pigs are the natural host for PEDV, it would be more appropriate to use pig cells as the screen cell lines. Thus, we conducted two screens for host factors essential for PEDV infection by constructing pig whole-genome KO libraries in IPEC-J2 and IPI-2I cell lines. We identified well-known essential host factors related to CoV replication, TMEM41B and TMEM106B (25–27), as well as a previously unreported host factor crucial for PEDV infection, YIPF5. Knockout of YIPF5 resulted in weakened PEDV infection. Further investigation into the stages of PEDV infection affected by the host factor revealed that YIPF5 primarily participates in the viral replication process. The absence of YIPF5 also reduced the infection of HCoV-OC43 and PDCoV. These findings suggest that YIPF5 may play a key role in the infection process of various CoVs.

The collaboration between host endomembrane systems and viral non-structural proteins is crucial for the formation of the CoV DMVs (29, 35). On the host side, DMV formation heavily relies on the involvement of host cellular membranes, particularly the ER membrane (32, 36–38). However, the specific host factors that participate in the formation of PEDV DMVs remain largely elusive. Previous studies have indicated that YIPF5 is a five-transmembrane protein, residing on the ER, the ER-Golgi intermediate compartment (ERGIC), and the cis-Golgi network. It is involved in maintaining the normal structure of the ER and Golgi apparatus, as well as regulating vesicular transport between these compartments (30, 31, 39). Based on these findings, we hypothesize that YIPF5 may play a pivotal role in the formation of DMVs. Double-stranded RNA (dsRNA), as a marker for single-stranded, positive-sense RNA viruses, resides in DMVs to evade the host innate immune response (40). Upon PEDV infection, YIPF5 was found co-localized with the dsRNA, suggesting a potential association. TEM results demonstrated that DMV formation was inhibited following YIPF5 knockout. The knockout of YIPF5 leads to the swelling of ER and the formation of stacked whorls, which is consistent with previous observations in YIPF5 siRNA-treated cells (30). Immunoelectron microscopy revealed that YIPF5 is involved in the early, intermediate, and mature stages of DMV formation, suggesting that it may participate in DMV formation through mechanisms such as membrane curvature and membrane closure.

Regarding the virus itself, the factors involved in the DMV formation are well established. The coronavirus nsp3, nsp4, and nsp6 play pivotal roles in the process of DMV formation (19, 41). Co-immunoprecipitation and confocal assays confirmed that YIPF5 interacts with nsp3, nsp4, or nsp6, and that they can co-localize. Previous research has conclusively shown that YIPF5 engages in interactions with SARS-CoV-2 nsp3 and nsp4 (42), hinting at its involvement in the crucial formation of SARS-CoV-2 DMVs. During SARS-CoV-2 infection, nsp6 provides essential structural support for viral replication by regulating the formation and organization of DMVs (20). The interaction between nsp6 and YIPF5, along with YIPF5’s role in the formation of clustered DMVs, indicates that YIPF5 may contribute to the formation of clustered DMVs via its interaction with nsp6. In WT IPEC-J2 cell lines, the co-expression of nsp3 and nsp4 resulted in the formation of circular, double-membrane proﬁles that were smaller than typical DMVs, consistent with previous findings from studies on SARS-CoV-2 and MERS-CoV (22, 43). In YIPF5 KO IPEC-J2 cell lines, the number of DMVs formed by nsp3 and nsp4 was significantly reduced, and a zippered structure in proximity to ER can be observed. The zippered structure may result from the misfolding of nsp3, nsp4, or nsp3/4. In YIPF5 KO cells, the co-localization and interaction between nsp3 and nsp4 were reduced, a phenomenon also observed in other host factors associated with DMVs, such as TMEM41B (22). Although our findings suggest that YIPF5 influences DMV formation by modulating the interaction between nsp3 and nsp4, the precise mechanisms by which YIPF5 regulates this interaction remain to be further explored in future investigations.

In summary, our study underscores the essentiality of YIPF5 as a critical host factor facilitating PEDV infection. YIPF5 interacts with PEDV nsp3, nsp4, and nsp6, thereby contributing to the formation of DMVs essential for the virus’s replication stage (Fig. 7F). These findings not only elucidate the intricate interplay between host factor and viral proteins during PEDV infection but also identify YIPF5 as a promising drug target for PEDV prevention and treatment strategies.

## MATERIALS AND METHODS

### Plasmid construction

The plasmid, pDX10-EF1α-Cas9-Puro, was generated by modifying the pEF1a-GFP-Puro vector, where the original GFP cassette was replaced with the Cas9 gene to facilitate selection and expression of Cas9 under the EF1α promoter. For targeted gene editing, sgRNA-expressing plasmids were derived from the pCRISPR-sg6 backbone (44). The sgRNAs were designed for YIPF5 using the CHOPCHOP tool. The designed oligonucleotides were synthesized, annealed, and subsequently inserted into the BbsI site of the pCRISPR-sg6 vector. The recombinant plasmids, pDX13-YIPF5-Flag and pDX13-GFP-YIPF5, were cloned into the pDX13-EF1α-Neo vector, which was linearized with NheI and NotI by Gibson Assembly Master Mix (New England Biolabs).

To ensure a high-level expression of the PEDV nsps in cells, the coding sequences of nsp3, nsp4, and nsp6 were codon optimized and cloned into the pCAGGS vector by General Biol company. Additionally, HA or Myc tag was fused to the N-terminus of each nsp to facilitate detection and functional analyses. All primers used are listed in Table S3.

### Cell culture and electroporation

The IPEC-J2, IPI-2I, Vero E6, and HEK293T cell lines were routinely maintained in our laboratory, and ACE2-Vero E6 cells were kindly provided by Prof. Yuhai Bi from the University of Chinese Academy of Sciences. All cell lines were confirmed to be free of mycoplasma contamination. The IPEC-J2, IPI-2I, Vero E6, ACE2-Vero E6, and HEK293T cells were propagated in Dulbecco’s Modified Eagle’s Medium (DMEM, Invitrogen) supplemented with 10% fetal bovine serum (FBS, Gibco). All cell lines were incubated at 37°C with 5% CO_2_.

For electroporation, the Nucleofector 2b Device (Lonza) was used according to the manufacturer’s instructions. The specific electroporation programs were optimized for each cell type: U031 for IPEC-J2 and T030 for ACE2-Vero E6. Prior to electroporation, endotoxin-free plasmids were prepared to minimize potential cellular toxicity and were mixed with prewarmed Nucleofector Solution to ensure efficient delivery into the target cells.

### Viruses

The PEDV CV777 strain (GenBank accession number: AF353511.1) and the PEDV LJX strain (GenBank accession number: MK252703.1) were kindly provided by Prof. Guangliang Liu from the Lanzhou Veterinary Research Institute, Chinese Academy of Agricultural Sciences. PEDV was propagated in Vero E6 cell lines. PDCoV was kindly provided by Dr. Kunli Zhang from the Institute of Animal Health, Guangdong Academy of Agricultural Sciences. HCoV-OC43 was kindly provided by Prof. Yuhai Bi from the University of Chinese Academy of Sciences. The experiments involving the infection of cells with HCoV-OC43 were conducted within the biosafety level-3 laboratory located at the University of Chinese Academy of Sciences following standard operating procedures.

### Plasmid library construction

The pig genome-scale CRISPR/Cas9 KO library was constructed by designing and synthesizing a total of 136,106 sgRNAs targeting genes in the Sscrofa11.1 genome assembly, along with 2,000 non-target sgRNAs as controls. These sgRNAs were subsequently amplified and inserted into the plentiCRISPR v2 vector (Addgene plasmid #52961) at the BsmBI site using the Gibson Assembly method (New England Biolabs). Subsequently, the ligation products were electroporated into DH10B competent cells, and the bacteria were harvested for maxipreparation with the Endo-free Plasmid Maxi kit (Qiagen).

### Lentivirus library production

For lentivirus library production, a total of 10 µg of the pig genome-scale CRISPR/Cas9 KO library plasmids were co-transfected with packaging plasmids (5 µg of pMD2.G, Addgene, #12259, and 10 µg of psPAX2, Addgene, #12260) into HEK293T cells seeded in 100 mm dishes using LentiFit transfection reagent (HANBIO, #HB-LLF-1000) following the manufacturer’s protocol. After 48 and 72 h of transfection, the cell supernatants containing the lentiviral particles were collected, filtered through a 0.45 µm low-protein binding membrane (Millipore, #HAWP04700) to remove cell debris, and then concentrated by adding 1/4 volume of a solution containing 25% PEG8000 and 0.75 M NaCl. The mixture was rotated and agitated at a low speed on a suspension instrument for 18 h at 4°C. Following centrifugation at 4,000 rpm for 15 min at 4°C, the supernatant was discarded, and the lentivirus pellets were gently resuspended in a harvest medium consisting of 70% DMEM, 10% FBS, 10% 4-(2-hydroxyethyl)-1-piperazineethanesulfonic acid (HEPES), and 10% bovine serum albumin (BSA). The resuspended lentiviruses were then aliquoted and stored at −80°C for future use.

### Cell library production and PEDV screens

The pig genome-scale CRISPR/Cas9 KO library was generated by infecting a total of 1 × 10^8^ IPEC-J2 or IPI-2I cell lines with genome-scale sgRNA library lentiviruses at an MOI of 0.3 in the presence of 10 µg/mL polybrene (Sigma-Aldrich, #TR-1003). After 3 d of 1 µg/mL puromycin selection to eliminate uninfected cells, the surviving cells were allowed to recover for an additional 7 d. Deep sequencing analyses revealed that the sgRNA coverage in IPEC-J2 and IPI-2I KO library cell lines was 87.11% and 86.54%, respectively, while the corresponding gene coverage achieved 99.97% for both cell lines. For genome-scale CRISPR screens, the pig genome-scale CRISPR/Cas9 KO library was divided into portions, each containing approximately 3 × 10^7^ cells. One portion served as a negative control, while three additional portions were used to conduct repeated screening experiments.

The pig genome-scale CRISPR/Cas9 KO library was infected with PEDV CV777 in DMEM supplemented with 10 µg/mL trypsin and was incubated at 37°C and 5% CO_2_. For IPEC-J2 KO libraries, three sequential rounds of PEDV CV777 infection with escalating MOIs (0.01, 0.1, and 1) were implemented. Briefly, after 3 d of PEDV infection, the surviving cells were collected and cultured for an additional 4 d before undergoing the next round of infection. After three rounds of screening, the third-round surviving cells were harvested and subsequently amplified for sgRNA sequencing. The IPI-2I KO library cell lines were challenged with PEDV CV777 at an MOI of 0.3 or 3. After 3 d of infection, the surviving cells were collected and expanded for deep sequencing analyses.

### Illumina sequencing of sgRNAs in the pig genome-scale CRISPR/Cas9 KO library cells

Genomic DNA was extracted from the surviving pig genome-scale CRISPR/Cas9 KO library using an animal tissue/cell genome DNA extraction kit (Solarbio, #D1700). The sgRNA region was specifically amplified by PCR using Q5 Hot Start High-Fidelity DNA Polymerase (NEB, #M0493). Following purification, the PCR products were sequenced on the Illumina HiSeq TM4000 platform. The sequencing data were analyzed using the MAGeCK package, which incorporates *Z*-scores into its analyses. All primers used are listed in Table S3.

### Generation of candidate gene knockout cell lines

To generate knockout cell lines targeting YIPF5, two paired sgRNAs were designed. The targeting sites of these sgRNAs encompass a significant portion of the exons of the YIPF5 gene, intended to induce large sequence deletions. Approximately 1 × 10^6^ cells were electroporated with 3 µg of pDX10-EF1a-Cas9-Puro plasmid, 1 µg of pCRISPR-sg6-TS1 plasmid containing the first sgRNA, and 1 µg of pCRISPR-sg6-TS2 plasmid carrying the second sgRNA, using the Nucleofector 2b Device (Lonza) according to the manufacturer’s instructions. Following 24 h of transfection, cells were treated with 1 µg/mL of puromycin for 3–4 d. Subsequently, single-cell clones were picked and genotyped. All primers used are listed in Table S3.

### RT-qPCR

Total RNA from cells and viral RNA from cells were extracted using TRIzol Reagent (Invitrogen, #15596018CN). Complementary DNAs (cDNAs) were synthesized from the extracted RNA using TransScript II All-in-One First-Strand cDNA Synthesis SuperMix for qPCR (Transgen, catalog number #AH341-01) in a total reaction volume of 10 µL. For each RT-qPCR reaction, 100 ng of cDNA and 5 nM of primer pairs were used with SYBR Green Mix (Takara, #CN830A). The reactions were monitored using the QuantStudio 3 System, programmed with one cycle of 5 min at 95°C, followed by 40 cycles of 10 s at 95°C and 32 s at 60°C. The relative expression levels were calculated using the 2^(^-ΔΔCt^) method, with the GAPDH gene serving as the normalization control. All primers used are listed in Table S3.

### Virus titers

Virus titers were performed as follows: confluent monolayers of control and YIPF5 KO cell lines in 12-well plates were inoculated in triplicate with PEDV at an MOI of 1. The cell culture supernatants were harvested at 48 h post-infection, and the virus titers were determined by the TCID_50_ assays.

### Viral attachment, internalization, and replication

#### Viral attachment

WT and YIPF5 KO IPEC-J2 cell lines were plated in 24-well cell culture plates. When the cells reached approximately 80% confluency, they were infected with a virus at an MOI of 5. The plates were then placed at 4°C for 2 h to allow for viral attachment without internalization. Then, the cells were washed three times with cold phosphate-buffered saline (PBS) to remove any unattached viruses. Subsequently, RT-qPCR was performed to quantify the amount of viral genomic RNA (gRNA). Additionally, immunofluorescence was used to detect the presence of viral N protein on the cell surface, indicating viral attachment.

#### Viral internalization

WT and YIPF5 KO IPEC-J2 cell lines were plated in 24-well cell culture plates. Once the cells reached approximately 80% confluency, they were infected with a virus at an MOI of 10. The plates were then placed at 4°C for 2 h to allow for viral attachment. After this initial attachment period, the cells were washed three times with cold PBS to remove any unattached viruses. Following the washes, DMEM was added, and the plates were transferred to a 37°C incubator for 1 h to allow for viral internalization. After this incubation period, the cells were washed three times with a phosphate solution of pH 1.5 to remove any viruses that had attached but not internalized. The cells were then washed three more times with PBS. Subsequently, RT-qPCR was performed to quantify the amount of viral gRNA within the cells. Additionally, immunofluorescence was used to detect the presence of viral N protein within the cells, indicating successful viral internalization.

#### Viral replication

WT and YIPF5 knockout cell lines were plated in 24-well cell culture plates. Once the cells reach approximately 80% confluency, they were infected with a virus at an MOI of 1. After 2 h of infection, the cells were washed three times with PBS to remove any unbound virus particles. DMEM was then added, and the cells were incubated at 37°C for 24 h to allow for viral replication. Following this incubation period, the cells were washed three times with PBS. Subsequently, RT-qPCR was performed to quantify the amount of viral gRNA within the cells, which reflected the level of viral replication. Additionally, immunofluorescence was used to detect the presence and distribution of viral dsRNA within the cells, providing further insights into the viral replication process.

### Immunofluorescence assay

WT and YIPF5 KO IPEC-J2 cell lines were seeded on coverslips and fixed with 4% paraformaldehyde for 10 min, permeabilized with 0.2% Triton X-100 for 10 min, and subsequently blocked with 2% BSA at room temperature for 1 h to reduce non-specific antibody binding. Subsequently, the cells were incubated with the primary antibodies: anti-PEDV N antibody (Medgen Labs, #SD-2, 1:1,000) or anti-dsRNA antibody (SCICONS, #10010200, 1:1,000) at 4°C overnight. After thorough washing to remove unbound primary antibodies, the cells were incubated with Alexa Fluor 555 donkey anti-mouse IgG (H+L) secondary antibody (Beyotime, #A0460, 1:500) at room temperature for 1 h to visualize the primary antibody binding. To stain the nuclei, the cells were incubated with 4′,6-diamidino-2-phenylindole (DAPI) at a final concentration of 10 µg/mL for 5 min at room temperature. Finally, the cells were visualized and photographed using a laser scanning confocal microscope (Zeiss).

### Western blotting

Cellular protein concentrations were determined utilizing the bicinchoninic acid (BCA) assay (Thermo Fisher Scientific, #A55860). Equal quantities of 20–30 μg of protein were loaded onto SDS-PAGE gels and subsequently transferred to polyvinylidene fluoride (PVDF) membranes. Following blocking and incubation with primary antibodies, including mouse monoclonal anti-PEDV N antibody (Medgen Labs, #SD-2, 1:1,000), rabbit polyclonal anti-YIPF5 antibody (Invitrogen, #PA5-67301, 1:250), rabbit polyclonal anti-Flag antibody (Proteintech, #20543-1-AP, 1:2,000), rabbit polyclonal anti-HA antibody (Proteintech, #66006-2-Ig, 1:2,000), rabbit polyclonal anti-Myc antibody (Proteintech, #16286-1-AP, 1:2,000), mouse monoclonal anti-GFP antibody (ShareBio, #SB-AB0005, 1:2,000), and mouse monoclonal anti-Tubulin antibody (Proteintech, #66031-1-Ig, 1:2,000), the membranes were incubated with the corresponding horseradish peroxidase (HRP)-conjugated secondary antibodies, specifically goat anti-mouse IgG (Proteintech, #SA00001-1, 1:2,000) or goat anti-rabbit IgG (Proteintech, #SA00001-2, 1:2,000). Protein bands were visualized using the Tanon 5200 imaging system.

### Transmission electron microscopy assay

WT, YIPF5 KO, and YIPF5 KO rescue IPEC-J2 cell lines were infected with PEDV at an MOI of 1 for 48 h. Following three washes with pre-cooled PBS, cells were fixed with 2 mL of 2.5% glutaraldehyde (Servicebio, #G1102) at room temperature for 30 min, and then at 4°C overnight to ensure complete fixation. Negative-stain electron microscopy was conducted by Servicebio company, and images were captured at high resolution using a transmission electron microscope (HITACHI, HT7700).

### Immunoelectron microscopy assay

Primary fixation was performed using a mixed fixative solution containing 4% paraformaldehyde and 0.5% glutaraldehyde. Membrane permeabilization was achieved with 0.1% Triton X-100, followed by blocking with a solution of 10% goat serum and 1% bovine serum albumin. The samples were then incubated with the primary antibody (rabbit IgG against YIPF5) and subsequently with the secondary antibody solution (goat anti-rabbit IgG conjugated with 10 nm colloidal gold). Fixation was carried out with 1% glutaraldehyde. After washing, gold enhancement was performed. The samples were washed again and fixed with 1% osmium tetroxide. Dehydration was achieved through a graded series of ethanol solutions. Infiltration and embedding were performed using SPI-Epon812 resin. Sections were prepared using an LKB-V ultramicrotome. Observation was conducted under a JEM-1200EX transmission electron microscope, with images recorded using a MORADA-G2 CCD camera.

### Co-immunoprecipitation assay

Immunoprecipitation (IP) was performed to validate the presence of specific proteins. HEK293T and IPEC-J2 cell lines were first transfected with the appropriate constructs, subsequently collected, washed thoroughly with PBS, and lysed in NP-40 lysis buffer. Following centrifugation to remove insoluble debris, the clarified lysate was incubated overnight at 4°C with gentle agitation in the presence of Protein G agarose beads pre-conjugated with specific primary antibodies. The immunoprecipitated proteins bound to the Protein G beads were then washed extensively with PBS to remove the unbound material and were eluted in 2× SDS-PAGE loading buffer by heating at 100°C for 5 min. Subsequently, the eluted proteins were subjected to SDS-PAGE electrophoresis, and the indicated proteins were analyzed by Western blotting to confirm their presence and/or interaction.

### Confocal microscopy

Confocal assays were performed to examine the co-localization of YIPF5 with dsRNA in IPEC-J2 cell lines, YIPF5 with PEDV nsp3, nsp4, or nsp6 in HEK293T cells, and the co-localization of YIPF5 with PEDV nsp3 and nsp4 in IPEC-J2 cell lines. For the first assay, IPEC-J2 cell lines were infected with PEDV at an MOI of 1 for 48 h. Cells were fixed and immunolabeled with primary antibodies against YIPF5 (Invitrogen, #PA5-67301, 1:250) and dsRNA (SCICONS, #10010200, 1:1,000). Subsequently, cells were imaged to identify double-fluorescent positive cells, indicating the co-localization of YIPF5 and dsRNA. For the second assay, HEK293T cells were co-transfected with YIPF5-Flag and either HA-nsp3, HA-nsp4, or HA-nsp6 for 24 h. Following transfection, cells were fixed and immunolabeled with primary antibodies specific to the Flag tag (Proteintech, #20543-1-AP, 1:500) and the HA tag (Proteintech, #66006-2-Ig, 1:500). For the third assay, HEK293T cells were co-transfected with GFP-YIPF5, HA-nsp3, and Myc-nsp4 for 24 h. Following transfection, cells were fixed and immunolabeled with primary antibodies specific to the Myc tag (Proteintech, #16286-1-AP, 1:500) and the HA tag (Proteintech, #66006-2-Ig, 1:500). After that, the cells were incubated with Alexa Fluor 555 donkey anti-mouse IgG (H+L) secondary antibody (Beyotime, #A0460, diluted 1:500), Alexa Fluor 405 donkey anti-rabbit IgG (H+L) secondary antibody (Beyotime, #A0605, diluted 1:500), or Alexa Fluor 488 donkey anti-rabbit IgG (H+L) secondary antibody (Beyotime, #A0423, diluted 1:500) at room temperature for 1 h to visualize the primary antibody binding. To stain the nuclei, the cells were incubated with DAPI at a final concentration of 10 µg/mL for 5 min at room temperature. Images were captured using a laser scanning confocal microscope (Zeiss) to visualize the co-localization.

### Data analyses

*P* values were determined by two-sided Student’s *t*-test. *P < 0.05, **P < 0.01, ***P < 0.001, and ****P < 0.0001. Data are representative of at least three independent experiments.

## Data Availability

All data supporting the findings in the paper are available within the paper and its supplemental material. All relevant data are available from the authors.

## SUPPLEMENTAL MATERIAL

Figure S1 jvi.00320-25-s0001.tif *Z* score analyses in the third round of PEDV screens in IPEC-J2 KO library.

Figure S2 jvi.00320-25-s0002.tif Presentation of Sanger sequences in YIPF5 monoclonal knockout cell lines and proliferation of WT and YIPF5 KO IPEC-J2 cell lines.

Figure S3 jvi.00320-25-s0003.tif Overexpression of YIPF5 in IPEC-J2 cell lines and re-expression of YIPF5 in YIPF5 KO IPEC-J2 cell lines.

Figure S4 jvi.00320-25-s0004.tif YIPF5 is an important host factor for HCoV-OC43 and PDCoV.

Supplemental legends jvi.00320-25-s0005.docx Legends for supplemental material.

Table S1 jvi.00320-25-s0006.xlsx Sequencing results of genes and sgRNAs in the third round of IPEC-J2 KO library screen.

Table S2 jvi.00320-25-s0007.xlsb Sequencing results of genes and sgRNAs in the IPI-2I KO library screen.

Table S3 jvi.00320-25-s0008.xlsx Primer pairs and sgRNA targeting sequences used in this study.

ASM does not own the copyrights to Supplemental Material that may be linked to, or accessed through, an article. The authors have granted ASM a non-exclusive, world-wide license to publish the Supplemental Material files. Please contact the corresponding author directly for reuse.
