# Supplementary figures and images for: YIPF5 is an essential host factor for porcine epidemic diarrhea virus double-membrane vesicle formation
Source: J Virol. 2025 May 27;99(6):e00320-25. doi: 10.1128/jvi.00320-25 (PMC12172457; doi:10.1128/jvi.00320-25)

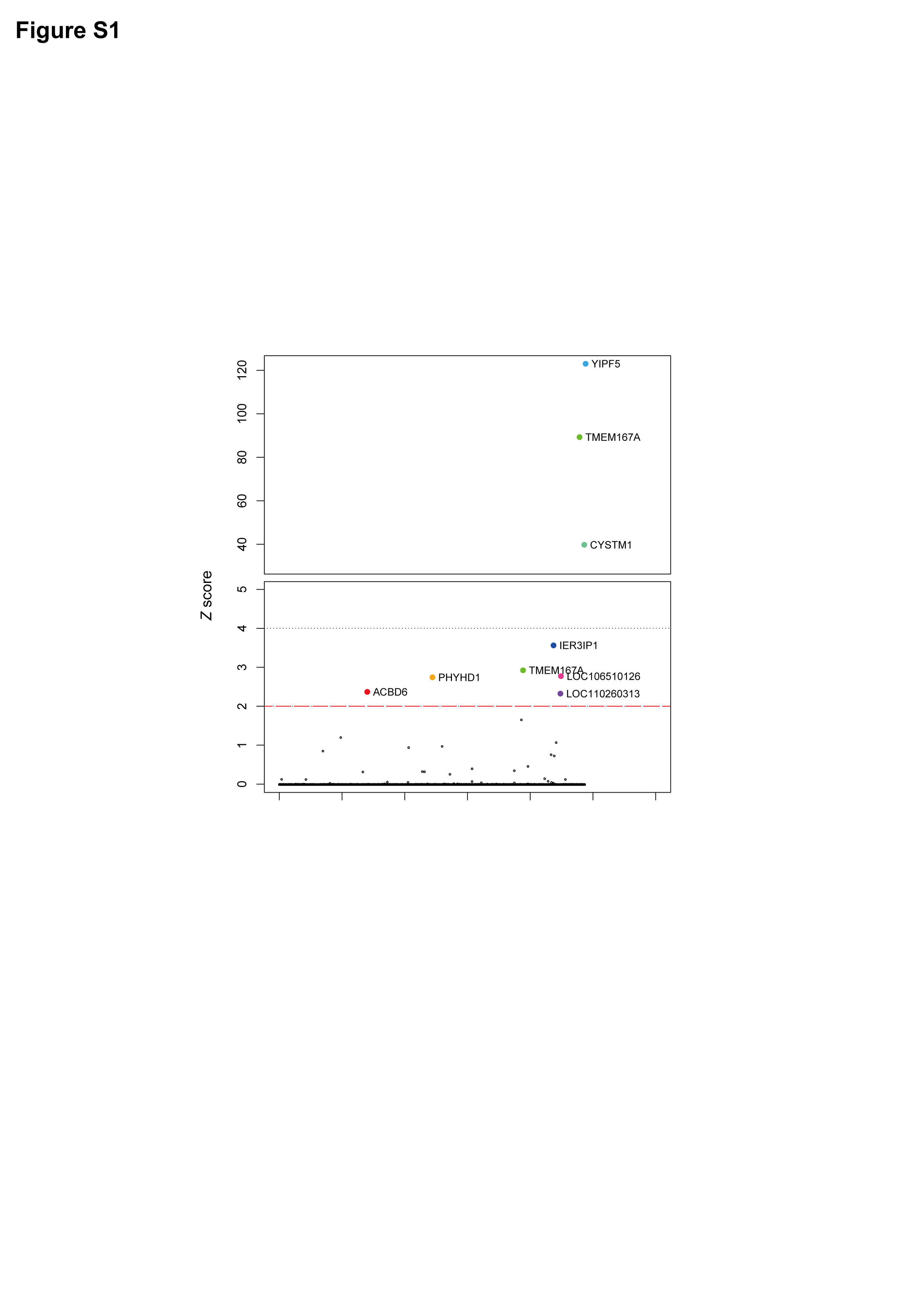

Supplement: Figure S1 — Z score analyses in the third round of PEDV screens in IPEC-J2 KO library. [file jvi.00320-25-s0001.tif]

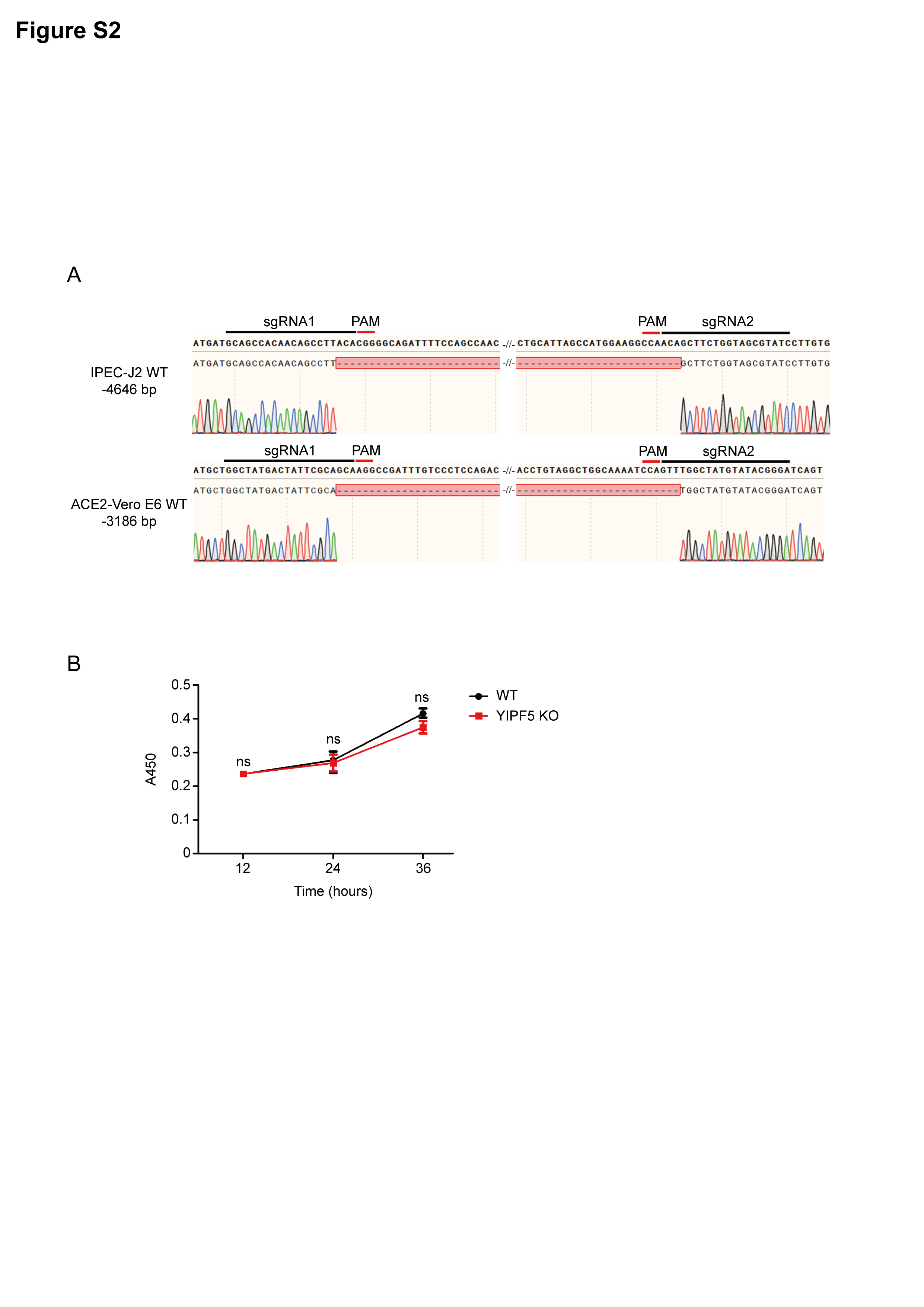

Supplement: Figure S2 — Presentation of Sanger sequences in YIPF5 monoclonal knockout cell lines and proliferation of WT and YIPF5 KO IPEC-J2 cell lines. [file jvi.00320-25-s0002.tif]

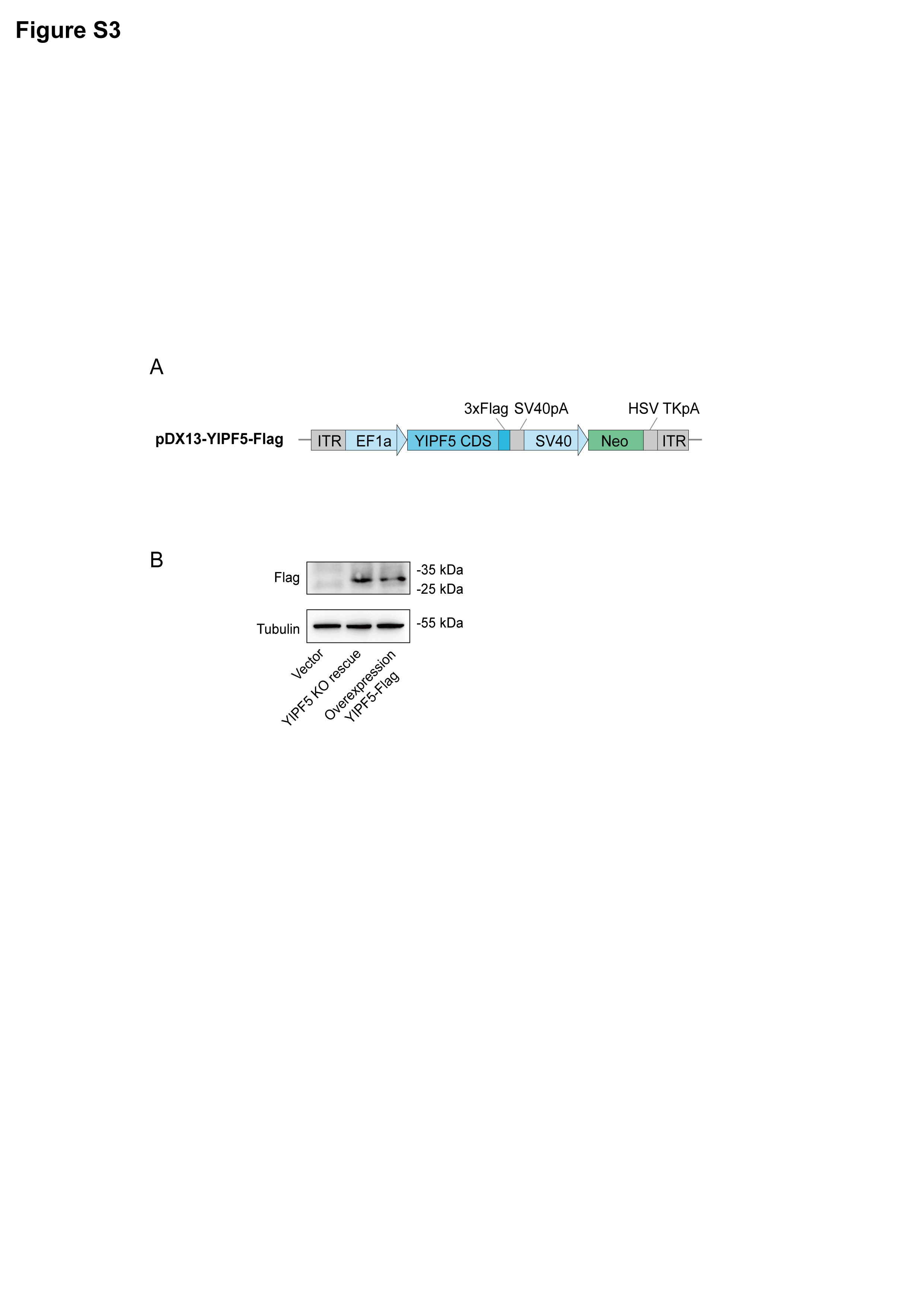

Supplement: Figure S3 — Overexpression of YIPF5 in IPEC-J2 cell lines and re-expression of YIPF5 in YIPF5 KO IPEC-J2 cell lines. [file jvi.00320-25-s0003.tif]

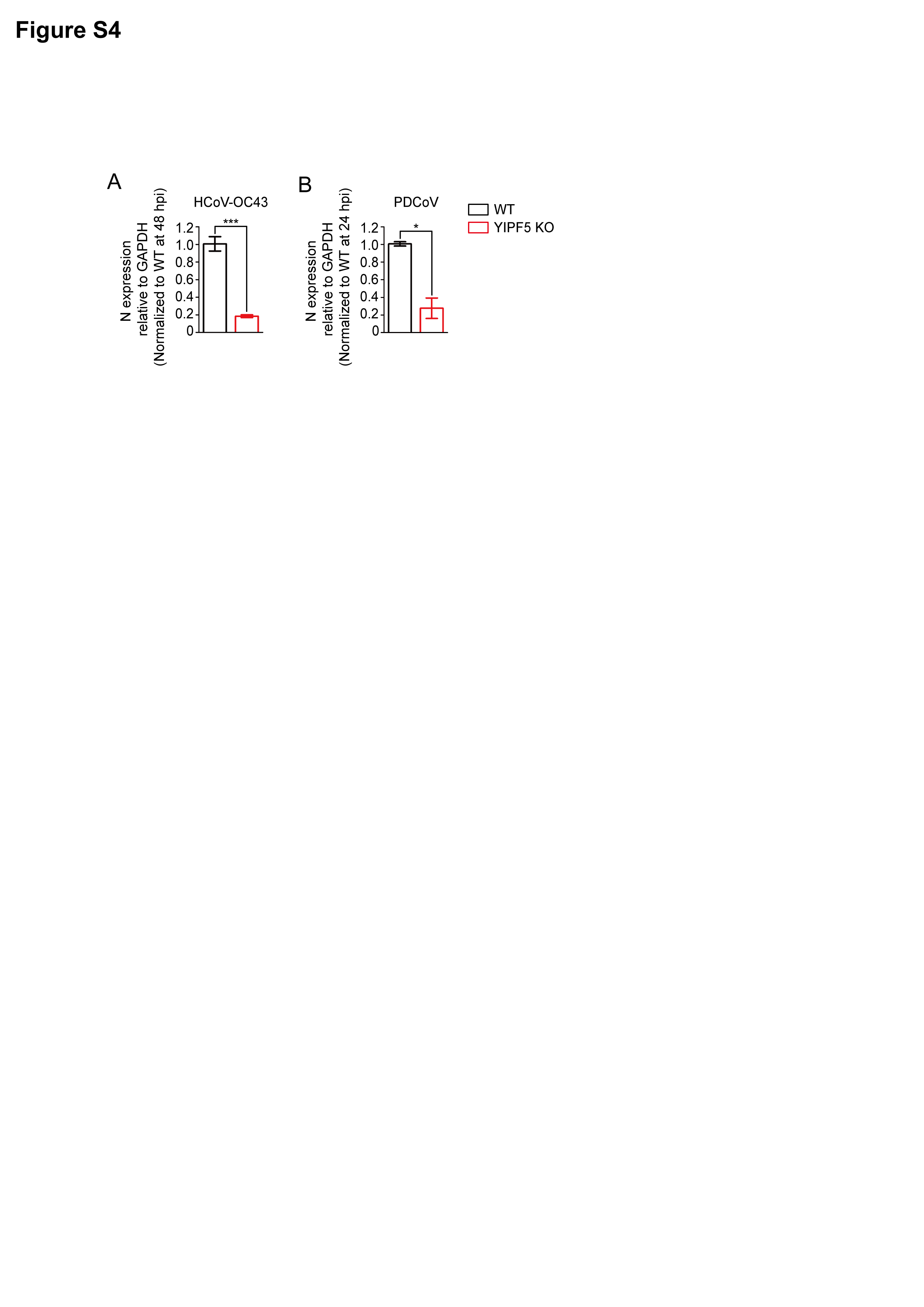

Supplement: Figure S4 — YIPF5 is an important host factor for HCoV-OC43 and PDCoV. [file jvi.00320-25-s0004.tif]
