# Supplementary material for: YIPF5 is an essential host factor for porcine epidemic diarrhea virus double-membrane vesicle formation
Source: J Virol. 2025 May 27;99(6):e00320-25. doi: 10.1128/jvi.00320-25 (PMC12172457; doi:10.1128/jvi.00320-25)
Supplement: Supplemental legends — Legends for supplemental material. [file jvi.00320-25-s0005.docx]

**Supporting information**

Fig. S1. *Z* score anaylses in the third round of PEDV screens in IPEC-J2 KO library. Red lines denote *Z* = 2.

Fig. S2. Presentation of Sanger sequences in YIPF5 monoclonal knockout cell lines and proliferation of WT and YIPF5 KO IPEC-J2 cell lines. (A) The Sanger sequencing results of YIPF5 KO IPEC-J2 and ACE2-Vero E6 cell lines. The underline indicates the deleted bases in the KO cells. The black line indicates the sgRNA. The red line indicates the PAM sites. (B) WT and YIPF5 KO IPEC-J2 cell lines were seeded into 96-well plates to evaluate cell proliferation by CCK8 assays at 12 h, 24 h, and 36 h. Abbreviations: sgRNA, small guide RNA; PAM, protospacer adjacent motif; WT, wild-type; bp, base pairs; ns, no significant.

Fig. S3. Overexpression of YIPF5 in IPEC-J2 cell lines and re-expression YIPF5 in YIPF5 KO IPEC-J2 cell lines. (A) Schematic illustration of the backbone of YIPF5-Flag plasmids. (B) Western blot assays for detecting the overexpression of YIPF5-Flag in IPEC-J2 cell lines and re-expression of YIPF5-Flag in YIPF5 KO IPEC-J2 cell lines. Tubulin served as the internal control protein.

Fig. S4. YIPF5 is an important host factor for HCoV-OC43 and PDCoV. RT-qPCR assay for determining the relative mRNA expression of HCoV-OC43 or PDCoV N protein in WT and YIPF5 KO Vero E6 cell lines (stably expressing the human ACE2) infected with HCoV-OC43 (A) at 48 h (MOI = 0.1) and PDCoV (B) at 24 h (MOI = 0.1). Abbreviations: WT, wild-type; KO, knockout; MOI, multiplicity of infection; N, nucleocapsid. * *P* < 0.05; *** *P* < 0.001. *P*-values were determined by two-sided Student’s t-test. Data are representative of at least three independent experiments.

Table S1 Sequencing results of genes and sgRNAs in the third round of IPEC-J2 KO library screen.

Table S2 Sequencing results of genes and sgRNAs in the IPI-2I KO library screen.

Table S3 Primer pairs and sgRNA targeting sequences used in this study.
